# Triplin: Functional Probing of Its Structure and the Dynamics of the Voltage-Gating Process

**DOI:** 10.3390/ijms232213765

**Published:** 2022-11-09

**Authors:** Marco Colombini, Kevin Barnes, Kai-Ti Chang, Muhsin H. Younis, Vicente M. Aguilella

**Affiliations:** 1 Department of Biology, University of Maryland, College Park, MD 20842, USA; 2 Regeneron Pharmaceuticals, Inc., Tarrytown, NY 10591, USA; 3 Department of Physics, Universitat Jaume 1, 12080 Castellon, Spain

**Keywords:** voltage dependence, voltage sensor, porin, prokaryote, rectification, trypsin, single channel, cooperativity, pore

## Abstract

Gram-negative bacteria have a large variety of channel-forming proteins in their outer membrane, generally referred to as porins. Some display weak voltage dependence. A similar trimeric channel former, named Triplin, displays very steep voltage dependence, rivaling that responsible for the electrical excitability of mammals, and high inter-subunit cooperativity. We report detailed insights into the molecular basis for these very unusual properties explored at the single-molecule level. By using chemical modification to reduce the charge on the voltage sensors, they were shown to be positively charged structures. Trypsin cleavage of the sensor eliminates voltage gating by cleaving the sensor. From asymmetrical addition of these reagents, the positively charged voltage sensors translocate across the membrane and are, thus, responsible energetically for the steep voltage dependence. A mechanism underlying the cooperativity was also identified. Theoretical calculations indicate that the charge on the voltage sensor can explain the rectification of the current flowing through the open pores if it is located near the pore mouth in the open state. All results support the hypothesis that one of the three subunits is oriented in a direction opposite to that of the other two. These properties make Triplin perhaps the most complex pore-forming molecular machine described to date.

## 1. Introduction 

The outer membrane of Gram-negative bacteria contains channel-forming proteins called porins. These membrane channels serve to select which molecules and ions reach the bacterial inner membrane. Some select mainly by excluding large structures that might harm the cell, such as enzymes and detergent micelles, whereas others are more selective in what they translocate [1,2]. Some porins display weak voltage dependence [3,4,5] in that a large voltage change is needed to convert the pores from an open to a closed state. That could be due to the conformation leading to pore closure and being coupled to the translocation of only a small amount of charge through the electric field across the membrane. When present, the voltage gating generally takes place at high transmembrane voltages, typically above 100 mV (for example [4,6]). These values are physiologically achievable by the development of Donnan potentials across the outer membrane when bacteria are in dilute salt solutions [7]. An exception to the requirement of high voltages is the porin called PorB (class 3) from Neisseria meningitides that gates at very low voltages: 50% channel closure at 15 mV and 25 mV depending on the sign of the applied voltage. Despite the low voltage needed for gating, PorB has a weak voltage dependence in that the estimated number of charges translocating through the transmembrane electric field is 1.5 [8]. Recently a novel pore-forming structure isolated from *E. coli* was described and named Triplin [9]. Table 1 shows the properties of Triplin that resemble those of well-studied porins [1,10]. Many porins are homotrimeric, and Triplin forms three pores whose conductance and ion selectivity are virtually identical but differ in their response to voltage. Two pores close at positive potentials and the last at negative potential. This was interpreted as indicating that one pore might be oriented in the opposite direction to the other two [9]. Crystals structures of homotrimeric porins show that all the pores are oriented in the same direction [1]. Another important difference is that Triplin displays very steep voltage dependence (an estimated 14 charges translocating through the electric field), very similar to that of the sodium and potassium channels responsible for the electrical excitability of mammalian neurons and muscles. Triplin also displays a high inter-subunit cooperativity and distinctly different behavior of the three functional pore-forming subunits. These properties are very different from those of any porin described to date. In the previous publication, the authors searched the *E. coli* protein database for any protein, with sequence similarity to OmpF and OmpN porins, that seemed to have the capacity to form pores but were able to isolate Triplin from all cells with knock-outs of each of the candidate proteins. Their conclusion is that despite the similarity to porins, Triplin may be a member of an as yet unidentified pore-forming protein family.

The research results presented here were obtained from electrophysiological recordings of single or a few Triplins reconstituted into planar phospholipid membranes. In order to place the results into an understandable framework, it is necessary to briefly describe the basic properties of Triplin as published [9]. The cooperativity and voltage-gating properties are complex and best-explained using a model constrained by the results of electrophysiological studies. At this point, the published model will be used, but in the Discussion section an updated model will be presented to include the new information.

**Figure 1:**
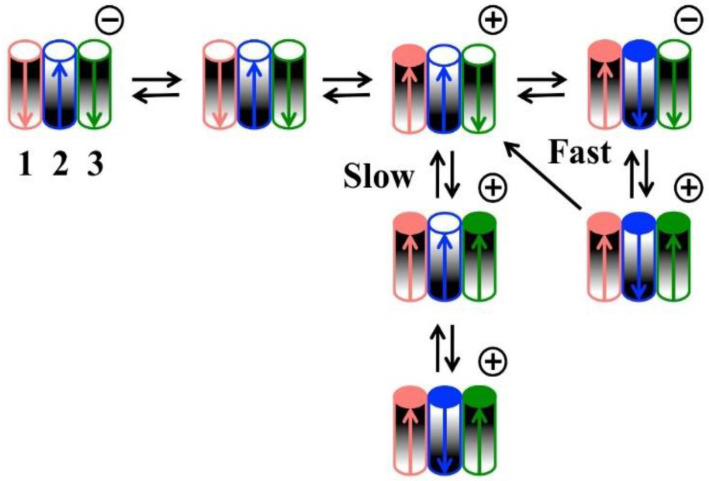
Model of the voltage gating of Triplin, as published in [9].

The model (Figure 1) depicts Triplin as a unit consisting of three identical cylindrical pore-forming subunits organized in a linear fashion to more easily account for the cooperative behavior. The subunits are labeled 1, 2, and 3 because each shows a distinct behavior. The grey shading of the subunits indicates the physical orientation of each subunit. The arrows indicate the dipole moment of the voltage sensor in each subunit. An open circle on the top of each cylinder indicates an open pore, and a filled circle indicates a closed pore. The positive and negative signs above the unit are the signs of the applied voltages. Note that the closure of one pore results in the realignment of the voltage sensor dipole with the electrical field but does not change the structural orientation. The subunits have strong positive cooperativity attributed to the dipole–dipole interactions. Due to the antiparallel structure, the subunits prefer to be in the same conformation, i.e., all open or all closed. When subunit 1 closes in response to an applied positive potential, the dipole–dipole stabilization between subunits 1 and 2 is eliminated and replaced by a dipole–dipole destabilization. This allows subunit 2 to respond to voltage and close as well. Likewise, subunit 3 gating only takes place after the closure of subunit 2.

**Figure 2:**
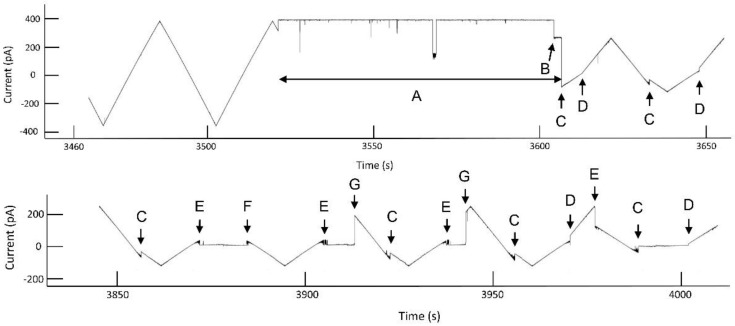
Voltage gating in a single Triplin. On the left side of the upper trace, a triangular voltage wave (+88/−87 mV; 30 mHz) results in no channel gating. In region “A”, a +90 mV potential was applied to the *cis* compartment. At point “B”, channel 1 closed. The resumption of the triangular wave causes gating of channels 2 and 3. “C” indicates the location of channel 2 closures and “D” the locations of channel 2 reopening. “E” and “F” are the locations of channel 3 closure and reopening, resp. “G” indicates double reopenings of channels 2 and 3. At 3977 s there is an unusual closure of channel 3 (a forbidden transition) when channel 2 is still open. The region between times 3977 s and the end of the record is an unusual period during which only channel 2 is open and gating.

A typical experiment (Figure 2) shows the properties of Triplin summarized in the model. The current/voltage relationship of a single Triplin structure follows Ohm’s law with no gating behavior. A high positive potential (generally 70 mV or higher; 90 mV in region “A”) results in the closure of one subunit at point “B” that is labelled as subunit 1. If the high positive potential were to be maintained over many minutes, subunit 3 would close followed by subunit 2 (not shown here but previously described [9]). After subunit 1 closure, resuming the triangular voltage wave shows complex behavior. There are subunit-closing events at negative potentials “C” and subunit-closing events at positive potentials “E”. The opening events occurred at “D”, “F”, and “G”. Note that if subunit 2 opens then subunit 3 does not close at positive potentials (e.g., at 3650 s). Occasionally, subunit 2 remains closed for a period of time and then subunit 3 gates repeatedly as the applied potential becomes positive (region 3857 s to 3910 s). Very rarely, subunit 3 remains closed and subunit 2 gates alone (region 3977 s to the end of the record). The periods of time in which only one subunit is conducting are useful for recording the conductance properties of a single pore. Often subunit 2 and 3 reopen simultaneously (points “G”). Thus, subunit 1 always closes first, allowing subunit 2 to be able to close at negative potentials. Subunit 3 can only close following the closure of subunit 2 with rare exceptions.

This manuscript aims to elucidate the molecular mechanism responsible for Triplin’s unique properties. The experimental results lead to a molecular mechanism that differs substantially from reports of the molecular basis for voltage gating in porins, e.g., [4,11]. The proposed mechanism also differs markedly from the molecular basis for voltage gating described for other steeply voltage-gated channel formers. The experimental approach is to study, with electrophysiological techniques, the behavior of single Triplins reconstituted into phospholipid membranes. The reported steep voltage dependence of subunits 2 and 3 each require that a voltage sensor, containing 14 charges, translocate through the transmembrane electric field to account for the observed voltage dependence. By neutralizing or cleaving these charges with specific reagents, we identified both the nature of the charges (positive or negative) and the amino acid side chains contributing to the voltage sensor. Adding the reagent to one membrane surface or the other localized charges responsible for the voltage dependence and demonstrated their translocation across the membrane, resulting from transitions between the open and closed conformations. We also find evidence for the cooperative behavior by which the closure of one pore influences the ability of another to close. From the non-linear dependence of current flow though the pores on the applied voltage, we find evidence that pore 2 is oriented in the opposite direction to pores 1 and 3. Finally, we find evidence for the location of the charged sensor on the loop regions outside the cylindrical pore. These results are combined into a model for the molecular mechanism of voltage gating that is unique and in harmony with all experimental results presented here and those previously published. This model expands the diversity of known voltage-gating mechanisms.

## 2. Results

### 2.1. The Dynamics of the Voltage Sensor Probed by Succinic Anhydride Modification

**Figure 3:**
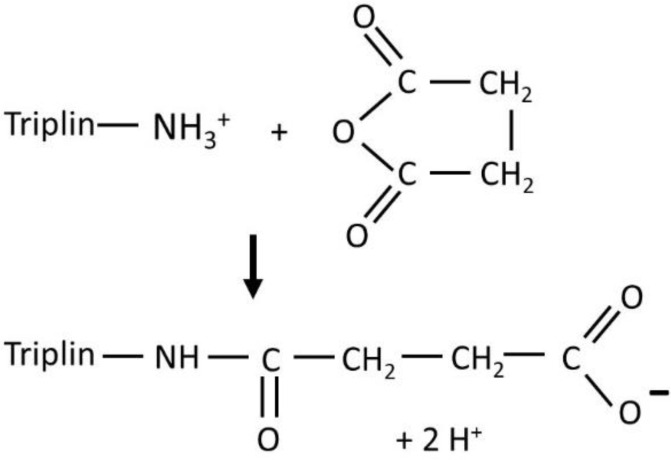
An illustration of the reaction of succinic anhydride with an amino group on Triplin.

In all steeply voltage-gated channels in which the voltage sensor has been identified, the voltage dependence of the conductance arises from the translocation of a charged domain through the transmembrane electric field, not necessarily transmembrane translocation. This provides the voltage-dependent energy change necessary for voltage gating. Thus, if the same is the case with Triplin, the modification of charged residues on the voltage sensor could either increase or decrease the voltage dependence of the conductance. Chemical modification with succinic anhydride was used to introduce carboxyl groups into the protein side chains. For example, a reaction with a lysyl side chain would produce an amide linkage and a free carboxyl group (Figure 3). If the sensor had a net negative charge, then this reaction would increase that charge by two units, and thus increase the steepness of the voltage dependence. For a sensor with a net positive charge, the opposite would be the result. Excess anhydride hydrolyzes to succinate in the aqueous solution in about 10 min.

#### 2.1.1. Succinic Anhydride Modification of the Voltage Sensor: Symmetrical Treatment

**Figure 4:**
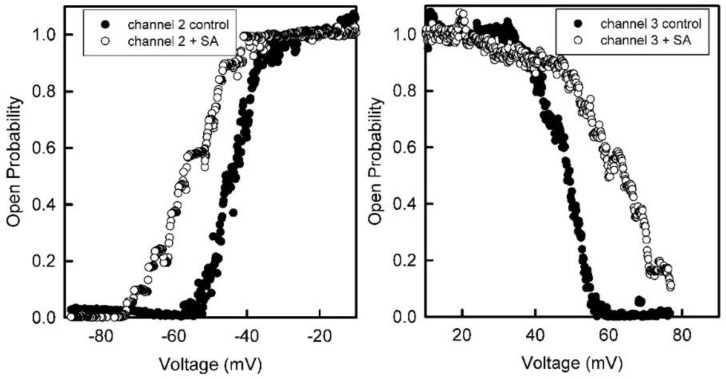
The voltage dependence of the probability of subunits 2 and 3 being in the open state before and after treatment with succinic anhydride (+SA) added to both sides of the membrane.

**Figure 5:**
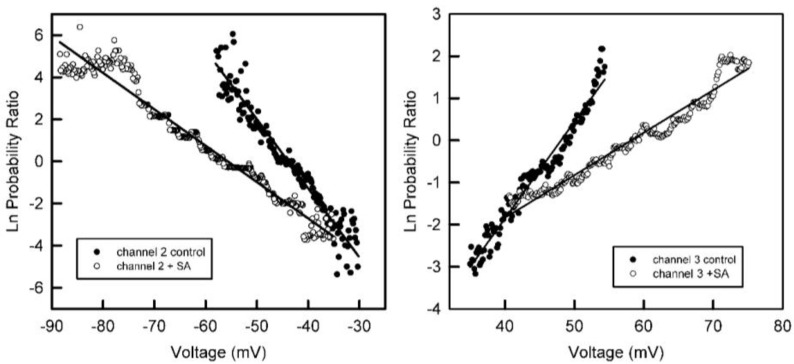
The data from Figure 4 were fitted to the Boltzmann 2-state distribution in order to obtain the voltage-gating parameters, *n* and *V*_0_. For subunit 2 that gates at negative potentials, the *n* value dropped from 8.4 to 4.8 and the *V*_0_ increased from −44 to −55 mV. For subunit 3 that gates at positive potentials, the *n* value dropped from 5.9 to 2.5 and the *V*_0_ increased from 46 to 70 mV after anhydride modification.

Addition of succinic anhydride to both sides of the membrane resulted in a reduction in the steepness of the voltage dependence (Figure 4). Fitting to the Boltzmann distribution yielded values of *n* about half the original values (Figure 5). This indicates that the net charge of the voltage sensor was reduced by half when amino groups were converted to carboxyl groups. Thus, the sensor must have had a net positive charge. However, because anhydride was added to both sides, the results do not specify where the sensor is located.

In the six experiments performed in which anhydride was added to both sides, the voltage dependence was never eliminated completely, much less reversed, as was expected if all the positive residues present were lysines. Of the two positively charged amino acid side chains on proteins, only lysines react with the anhydride. Thus, either most of the charges are due to arginine residues or most are not accessible to anhydride modification using the described methodology. The symmetrical addition of succinic anhydride increased Triplin’s preference for the passage of cations. A salt gradient of 1.0 M KCl vs. 0.10 M KCl resulted in a reversal potential for Triplin of −21 mV on the high salt side, which gives a permeability ratio of P+/P− = 3.0 favoring potassium ions, as previously reported [9]. Treatment with succinic anhydride increased the reversal potential to −31 mV, and thus P+/P− = 5.6. These values at a high salt concentration indicate a net negative charge in the inner walls of the pores, and this is increased by anhydride modification. Thus, the voltage sensor cannot be located within the pore itself but somewhere outside the pore. This is also the case for the Na+/K+/Ca+ channel family [12] and unlike the situation for VDAC channels [13].

#### 2.1.2. Voltage Sensor Location in Different States of Each Triplin Subunit Using One-Sided Anhydride Addition

**Figure 6:**
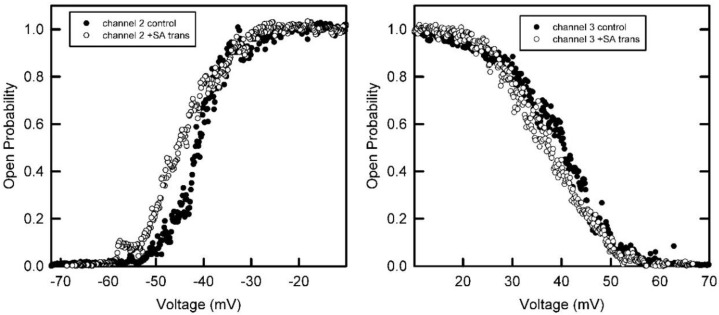
The voltage-dependence of the probability of subunits 2 and 3 being in the open state before and after treatment with succinic anhydride added only to the *trans* side of the membrane. The *n* value for subunit 2 dropped from 7.9 to 5.6, whereas for subunit 3 it did not change significantly (from 4.4 to 4.6).

The addition of succinic anhydride to only one side of the membrane resulted in a decrease in voltage dependent gating of subunit 2 when the anhydride was added to the *trans* side of the membrane only (Figure 6 left side). During the reaction, subunit 2 was in the open conformation. Thus, the voltage sensor for subunit 2 seems to be located on the *trans* side when subunit 2 is open. In contrast, in the same experiment, the addition of the anhydride did not alter the voltage dependence of subunit 3 (Figure 6 right side) and subunit 3 was also in the open-state conformation. Thus, the voltage sensor of subunit 3 was not exposed to the *trans* side when pore 3 was open.

**Figure 7:**
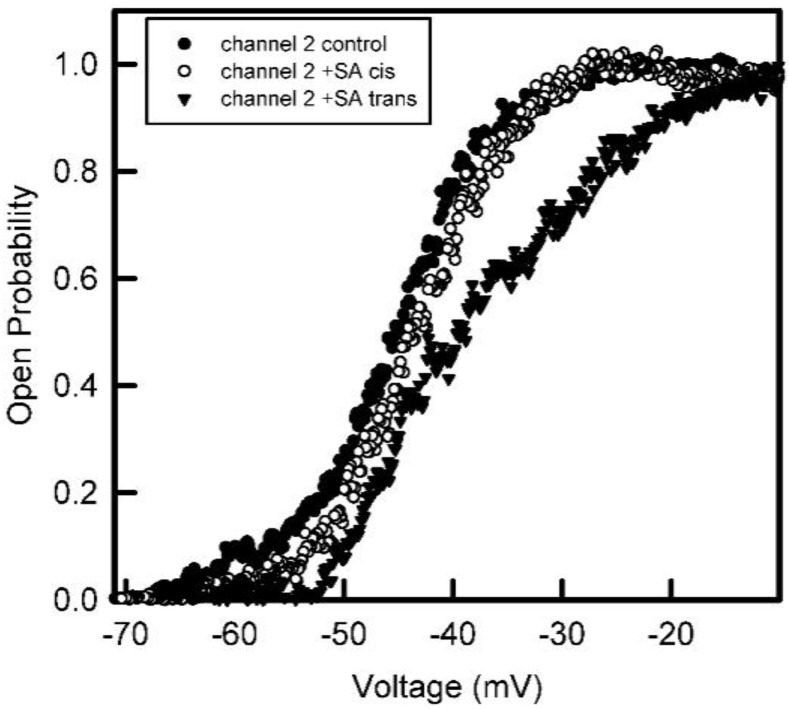
The voltage dependence of the probability of subunit 2 to be in the open state before and after treatment with succinic anhydride added first to the *cis* and then to the *trans* side of the membrane. Addition to the *cis* side resulted in no significant change in *n* (from 5.3 to 6.1) but addition to the *trans* compartment caused the *n* value to drop to 2.8.

In a separate experiment (Figure 7), anhydride was first added to the *cis* compartment (the side of protein addition) and subsequently the anhydride was added to the *trans* side. No significant change in the voltage dependence of subunit 2 was observed upon *cis* anhydride addition, but the voltage dependence decreased after anhydride addition to the *trans* side. Thus, the sensor of subunit 2 was only located on the *trans* side when the subunit was in the open state.

Unexpectedly, with one exception, the addition of succinic anhydride to the *cis* side of the membrane did not result in a decrease in the voltage dependence of subunit 3. Since subunit 3 closes when the *cis* side of the membrane is made positive, we expected that the positively charged sensor would be accessible from the *cis* side. Those experiments were done holding the transmembrane potential at 10 mV and subunit 2 was in the open conformation. Thus, we could not reliably detect the location of the voltage sensor for subunit 3 when pore 2 is open.

In order to probe the dynamics of the voltage sensor, anhydride was added while holding subunit 2 in the closed conformation by applying a high negative potential (typically −65 mV). The addition of anhydride to the *trans* compartment resulted in no change in the steepness of the voltage dependence of both subunits 2 and 3. The addition of anhydride to the *cis* compartment did reduce the steepness of the voltage dependence of subunit 2, indicating that the closure of subunit 2 was associated with the translocation of its voltage sensor from the *trans* to the *cis* side. This established the molecular basis for the voltage control of subunit 2. Interestingly, the voltage dependence of 3 was also reduced under these conditions. Thus, somehow the closure of subunit 2 exposes the voltage sensor of subunit 3 and it faces the *cis* compartment. Thus, the closure of pore 2 exposes the voltage sensor of pore 3, allowing it to respond and close when a positive voltage is applied.

Probing the dynamics of the voltage sensor of subunit 3 was very difficult because the high positive potential required for subunit 3 to be held in the closed state (~+50 mV), also resulted in subunit 2 opening. In the large majority of experiments, the slow reopening kinetics of subunit 2 were not slow enough to keep the subunit closed during the anhydride modification process. Subunit 2 opening forces subunit 3 to also open, thus, spoiling the attempt to modify the sensor of subunit 3 in the closed state. However, subunit 2 rarely remains in a closed conformation for extended periods of time, and, under those conditions, subunit 3 can be held in a closed state long enough to perform the needed experiments. Three experiments were achieved: two with a *trans* addition and one with a *cis* addition of anhydride. The steepness of the voltage dependence was reduced by adding anhydride to the *trans* compartment and not to the *cis* compartment. Thus, in the cases of both subunits 2 and 3, subunit closure is associated with the translocation of charge across the membrane, as expected from the energetics of the voltage-gating process.

Table 2 summarizes the results of the one-sided experiments performed and includes results of the Student’s *T*-test. The results show high statistical significance for the modification of the voltage sensor by the anhydride on the *trans* side when subunit 2 is open and on the *cis* side when it is closed. For subunit 3, anhydride modification was highly significant when the anhydride was added to the *cis* compartment and subunit 2 was held in the closed conformation. When subunit 2 is in the open conformation, then the sensor of subunit 3 is not accessible. Insufficient data were collected to allow for statistical testing for modification of the sensor of subunit 3 from the *trans* compartment when subunit 3 was in the closed conformation. Nevertheless, in two experiments performed with subunit 3 closed, the voltage dependence was reduced by anhydride addition to the *trans* compartment, indicating that the voltage gating of pore 3 involves the translocation of charge across the membrane, such as pore 2, but in the opposite direction.

### 2.2. Trypsin Cleavage of the Voltage Sensor Identifies Its Location When the Subunit Is in an Open or Closed State

**Figure 8:**
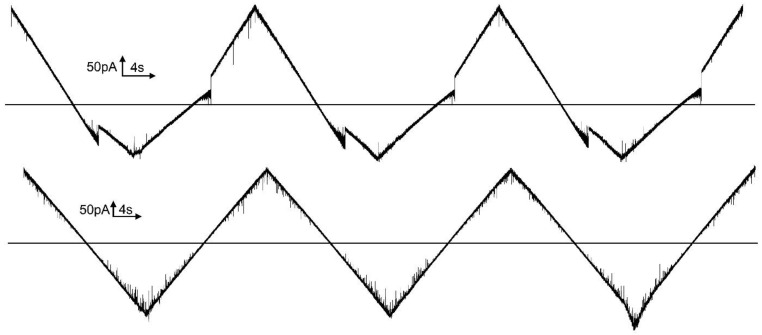
Trypsin added to the *trans* compartment cleaved the sensor of subunit 2. The upper trace was recorded before trypsin addition (triangular wave +81/−79 mV; 30 mHz). A constant 10 mV was applied followed by trypsin addition (60 ug to the *trans* compartment). After 10 min of trypsin treatment, 1 mg of soybean trypsin inhibitor was added to the *trans* compartment and the triangular voltage wave was resumed (lower trace).

**Figure 9:**
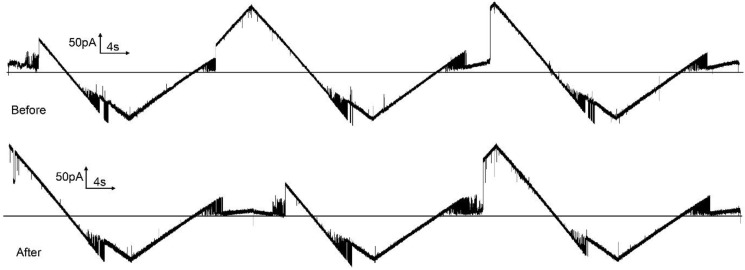
Trypsin failed to cleave the sensors of any subunits when added to the *cis* side when subunits 2 and 3 were in the open state. The upper trace was recorded before trypsin addition (triangular wave +80/−80 mV; 30 mHz). A constant 10 mV was applied followed by trypsin addition (40 µg to the *cis* compartment). After 10 min of trypsin treatment, 1 mg of soybean trypsin inhibitor was added to the *cis* compartment and the triangular voltage wave was resumed (lower trace). Subsequent addition of 40 µg trypsin to the *trans* compartment resulted in rapid reopening of subunit 2 and loss of voltage gating (not shown).

Trypsin is an endopeptidase that exhibits a high level of preference for cleaving peptide bonds at the c-terminal side of the basic amino acids, lysine, and arginine. The succinic anhydride experiments identify the voltage sensors of subunits 2 and 3 as being positively charged, including the presence of lysine residues. Thus, if accessible, the sensor should be cleaved by trypsin. With the loss of the voltage-dependent energy that favors subunit closure, the subunits should return to or remain in the open conformation. Trypsin addition to the *trans* compartment with subunit 2 in the open conformation resulted in subunit 2 being unable to gate and remaining in the open state (Figure 8). Exposure to trypsin was limited to 10 min by the addition of soybean trypsin inhibitor. Subunit 3 did not gate after the trypsin treatment because subunit 2 could not close. It is unclear from this experiment whether the sensor of subunit 3 was cleaved by this treatment. When the same experiment was performed, except with trypsin being added to the *cis* compartment (Figure 9), the gating of subunit 2 was unaffected, as well as the gating of subunit 3. Thus, as was the case with the anhydride modification, the sensor of subunit 3 is not accessible from the *cis* compartment when subunit 2 is in its open state.

**Figure 10:**
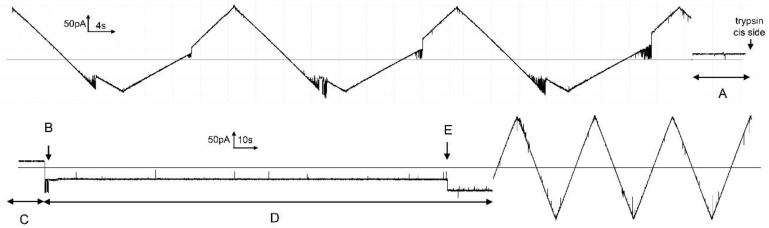
Trypsin cleavage of the sensor of subunit 2. The upper trace shows the gating of subunits 2 and 3 of a single Triplin in response to a triangular voltage wave (+/−80 mV). In region “A”, 10 mV was applied prior and during treatment with 20 µg trypsin added to the *cis* side. After 10 min of exposure to trypsin (region “C”), −36 mV was applied (region “D”). Subunit 2 closed almost immediately (“B”). At point “E”, subunit 2 opened and the subsequent application of a triangular voltage wave resulted in no gating.

In the experiment illustrated in Figure 10, trypsin was added to the *cis* compartment when subunit 2 was in the open state and then −36 mV was applied to close subunit 2. After 2.7 min, subunit 2 spontaneously opened, and the subsequent application of a triangular voltage wave showed that subunit 2 could no longer close. Again, this is in agreement with the sensor of subunit 2 being accessible to the *cis* compartment when subunit 2 is closed.

**Figure 11:**
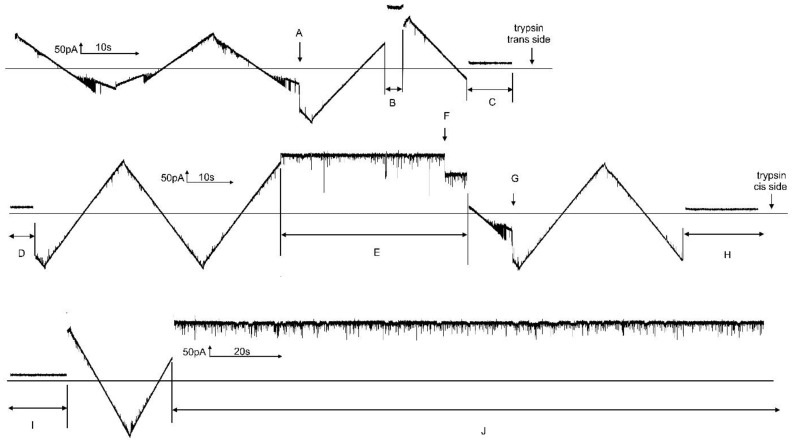
Trypsin cleavage of the sensor of subunit 1 from the *cis* side but not from the *trans* side. On the left side of the uppermost tracing, a single Triplin is responding to the applied triangular voltage wave (+81/−79 mV) with the closure of subunit 2 at elevated negative voltages. At point “A”, subunits 1 and 2 open essentially simultaneously, resulting in no gating. In region “B”, +102 mV was briefly applied followed by the resumption of the triangular voltage wave. In region “C”, + 10 mV was applied and 40 µg trypsin was added to the *trans* compartment. After 10 min of exposure to trypsin, 1 mg of soybean trypsin inhibitor was added. Region “D” is after the inhibitor addition. Resuming the triangular voltage wave resulted in no gating, indicating that subunit 1 was still open. In region “E”, +92 mV was applied and subunit 1 closed at point “F”. The triangular voltage wave was resumed and subunit 2 closed followed by reopening of subunits 1 and 2 at point “G”. In region “H”, the voltage was held at +10 mV followed by the addition of 40 µg of trypsin to the *cis* compartment. Trypsin was allowed to react for 10 min and region “I” is the end of that reaction time. The triangular voltage wave showed that subunit 1 was still closed. The application of +92 mV to try to close subunit 1 (region “J” and beyond) resulted in no closure of subunit 1.

The location and accessibility of subunit 1 were probed by trypsin treatment. In Figure 11, a single Triplin was gating normally but stopped when subunit 1 opened (A). The addition of trypsin to the *trans* side followed by trypsin inhibitor 10 min later did not eliminate the ability of subunit 1 to close following the application of +92 mV (F). Closure of subunit 1 allowed subunit 2 to gate, also demonstrating that the sensor of subunit 2 was not accessible from the *trans* side when subunit 1 was open. Following the reopening of subunit 1 (G), the addition of trypsin to the *cis* compartment and reacting for 10 min resulted in the inability of subunit 1 to close after a long and persistent application of +92 mV. Thus, in the open confirmation, the sensor of subunit 1 is accessible from the *cis* but not from the *trans* side.

### 2.3. Use of Rectification to Assess Subunit Orientation

Deviations from Ohm’s Law (rectification) have been reported for porins [14,15]. Weak rectification is also found in the pores formed by Triplin. This rectification likely results from some asymmetry in the effective fixed charge along the ion-conducting pathway, possibly a combined effect of the pore geometry and the net charge of ionizable residues. Such asymmetry resulted in a higher conductance with increasing negative applied voltages and lower conductance when increasing positive voltages. This negative slope, *dG*/*dV,* is qualitatively consistent with an excess net positive charge closer to the *cis* side (where voltage *V* is applied) or an excess negative charge closer to the *trans* side (electrically grounded). Unlike the motion of the voltage sensor, one would expect the conducting pathway to remain stationary with changes in transmembrane voltage, as long as the subunit remained in the open conformation. Thus, this rectification was exploited to test the hypothesis that the subunits are oriented in an antiparallel fashion.

Triplin-containing samples were dispersed only on the *cis* side of a solvent-free planar membrane made from phospholipid monolayers. Hundreds of experiments have been performed and, in all cases, Triplin inserts in only one direction based on the sign of the voltage needed to close each of the three subunits. Experiments were performed on membranes containing a single Triplin. Initially, the inserted Triplin is fully open, and thus the rectification is the result of the current flowing through all three subunits. Upon closure of subunit 1 at positive potentials, the rectification is only the result of the current flowing through subunits 2 and 3. For subunits 2 and 3, the rectification of each one alone could be obtained directly.

The conductance/voltage relationship in all cases was obtained from the current/voltage relationship by calculating the chord conductance. A linear fit was made separately to data collected at both positive and negative potentials in order to obtain a quantitative measure of the voltage dependence of the conductance. The gating of subunits 2 and 3 only allowed reliable measurements at positive voltages for subunit 2 and negative voltages for subunit 3.

**Figure 12:**
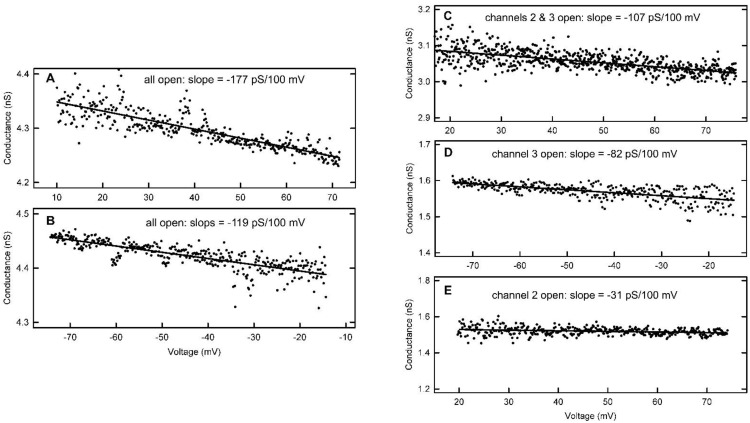
Examples of rectification recorded on single Triplin channels. The triangular voltage waves were run at 30 mHz. Panels (**A**,**B**) are examples of the voltage dependence of the conductance when all 3 pores are open in a single Triplin. Panels (**C**, **D** and **E**) are the same for pores 2 and 3 open, pore 3 only open and pore 2 only open respectively.

Examples of conductance/voltage plots are illustrated in Figure 12. Note that reliable measurements of the chord conductance could not be made at low voltages. The deviation from Ohm’s law was quantitated by linear regression analysis. Multiple measurements were made for each condition in each individual experiment, and the values for each condition were averaged. The results from different experiments are reported as an average with the SEM in Table 3.

The sign of the dependence of conductance on voltage was changed to reflect how the conductance changed with increasing electric field, positive in the negative voltage region.

The overall conductance increased as the transmembrane voltage was made more negative and decreased when the voltage was made more positive. However, to gain insight into the orientation of the individual subunits, it is necessary to obtain the rectification properties of each one in both the positive and negative-voltage regions. Assuming that when two or three subunits are conducting independently the voltage dependence of the conductance is additive, then from this data one can calculate the missing single subunit rectification values (Table 4).

The results indicate that subunits 1 and 3 display essentially identical rectification, consistent with the presence of an asymmetrical charge. The rectification exhibited by subunit 2 is quite different. Dependence of the conductance on transmembrane voltage is both much weaker and bell-shaped, declining with increasing electric field in both the positive and negative direction. This result does not fit with a simple anti-parallel subunit orientation with respect to subunits 1 and 3. However, when combined with the results obtained from probing the dynamics of the voltage sensor (next section), the situation becomes clarified.

## 3. Discussion

Triplin forms three pores through the membrane that have the same conductance and ion selectivity [9], and thus resemble homotrimeric porins. However, the pores formed by Triplin have steep voltage dependence, requiring an estimated translocation of charge across the membrane of 14 units [9]. This property requires a gating mechanism radically different from those proposed for the well-established porins, e.g., [11,16]. The most straight-forward mechanism would be to couple the conformational change responsible for pore opening and closure with the translocation of a charged domain (the voltage sensor) across the membrane. This voltage-dependent energy change would then drive the structural change.

### 3.1. Identification and Localization of the Voltage Sensors of the Three Pore-Forming Structures

Since charge translocation through the electric field across the membrane is necessary to achieve steep voltage dependence, what charged amino acid side chains might be responsible? If the voltage sensor were a positively charged structure containing lysine residues then succinic anhydride conversion of amino groups into carboxyl groups would reduce the charge on the sensor, and thus the steepness of the voltage dependence. That was indeed the case, thus, providing strong evidence for the positive charge of the voltage sensor and its accessibility to modification. Considering the cation selectivity of the pores, the highly charged sensor could not be located withing the ion-conducting pore but rather on the protein surface facing the bulk aqueous phase. The asymmetric anhydride addition showed that for pore 2, the voltage sensor could be modified from the *trans* side when pore 2 was open and from the *cis* side when channel 2 was closed, demonstrating that the sensor translocates across the membrane when pore 2 gates. Note that a negative potential on the *cis* side of the membrane closes pore 2 and also causes the positive sensor to move to the *cis* side. The two observations are in agreement. This agreement is expected and necessary and that charge motion must be coupled to the structural change of the pore-forming subunit, resulting in voltage-dependent gating of pore 2. For pore 3, the same result was obtained except that the sensor translocation was in the opposite direction, from the *cis* side to the *trans* side. The opposite direction of the motion of the sensor is in agreement with pore 3 gating when the *cis* compartment was made positive. These findings were supported by determining the accessibility of the sensor to cleavage by trypsin, a protease that specifically cleaves at lysine and arginine residues.

The inability to measure the voltage gating parameters for pore 1 meant that anhydride modification would not be useful to probe the motion of the sensor of pore 1. However, the ability of pore 1 to close was eliminated when trypsin was added to the *cis* side but not the *trans* side when pore 1 was open. Trypsin cleavage prevented pore 1 from closing, which is consistent with the conclusion that like pore 3, the gating of pore 1 was also associated with the translocation of its voltage sensor. Again, the direction of motion is consistent with the sign of the voltage needed to close the pore.

### 3.2. Mechanistic Insight into the Cooperativity among the Three Pore-Forming Structures

The experiments also provided mechanistic evidence for the influence of pore 1 on pore 2 and pore 2 on pore 3. The voltage sensor of pore 2 was not accessible to trypsin cleavage from the *trans* side until pore 1 was closed. This is consistent with pore 2 being unable to respond to voltage until pore 1 is closed. Similarly, the sensor of pore 3 was not accessible to anhydride modification from the *cis* side until pore 2 was closed. Again, this explains why pore 3 cannot respond to voltage until pore 2 is closed.

### 3.3. Relative Orientation of the Pore-Forming Subunits

The steep voltage dependence and the cooperativity are strongly supported by the succinic anhydride and trypsin experiments and these further support the conclusion that subunit 2 is oriented in the opposite direction to subunits 1 and 3. This conclusion is contrary to the structural information of all homotrimeric porins studied to date (for example, [1]). It is possible that despite essentially identical conductance and selectivity [9], the structure of subunit 2 is very different from that of 1 and 3 and that, physically, the subunits may have the same transmembrane orientation but different voltage sensor location. The rectification studies demonstrate that pore 1 and 3 have essentially indistinguishable rectification properties, whereas those of pore 2 are distinctly different. Although this finding could easily be interpreted as pore 2 just having a different structure despite being similar to the others in other ways, a careful, quantitative analysis favors the anti-parallel model.

**Figure 13:**
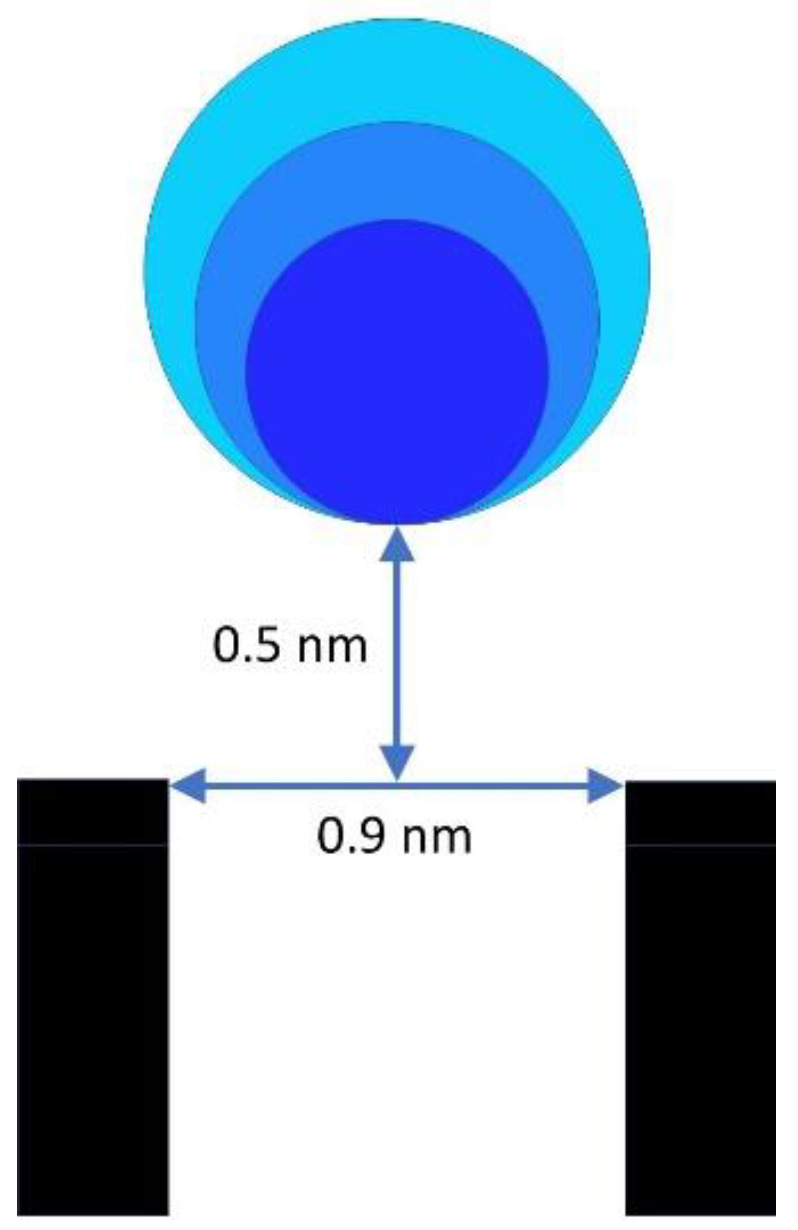
PNP calculations indicate that a sphere composed of 10 elemental positive charges whose lower edge is located 0.5 nm from the opening of a 0.9 nm diameter pore (5 nm in length) will produce a rectification of approximately 100 nS/100 mV in an aqueous medium containing 1 M monovalent salt. The spheres are 0.3, 0.4, and 0.5 nm in radius, each of which could produce the observed rectification. To save space, only a portion of the pore is illustrated.

One way of accounting for the rectification of pores 1 and 3 would be to have an asymmetric distribution of fixed negative charge along the conducting pathway. Given the cation selectivity of the pore, the charge would need to be negative and located near the *trans* side. Poisson–Nernst–Planck (PNP) calculations show that a fractional charge (0.2 e) spread over a 1 nm region near the *trans* side of a 5 nm long pore with a 0.9 nm diameter would result in the observed rectification. The problem with this proposal is that the selectivity of all the pores is virtually the same and yet the rectification is different. Thus, the charge on the walls of the pore that is responsible for the cation selectivity is unlikely to be the source of the rectification. However, the asymmetric charge could be outside the pore itself. A much larger charge could produce the same rectification if it were located on the external loops of a beta barrel structure, for example. What if the charge on the voltage sensor were responsible for both the voltage gating and the rectification, but not for the pore selectivity? A large positive charge near the mouth of the pore on the *cis* side (where the voltage sensor is located for pores 1 and 3) might work. PNP calculations were performed in order to determine whether a positively charged loop located outside the pore but close to it could be responsible for the observed rectification attributed to charge asymmetry. To simplify the calculation, a charged sphere with 10 elemental positive charges was located just outside the pore of a channel, 0.9 nm in diameter and 5 nm long. The aqueous solution consisted of 1 M monovalent salt as in the experiments performed. The size of the sphere and the distance from the pore opening were varied to determine conditions that would produce a rectification close to 100 pS/100 mV, essentially as observed experimentally. Figure 13 shows the outcome. Within a range of sphere radii of 0.3 to 0.5 nm, the distance of the edge of the sphere to the pore opening needed to be 0.5 nm. Thus, the conditions to achieve the observed rectification are consistent with what is possible with a beta barrel protein structure.

The weak decline in conductance with increasing voltage in both the positive and negative voltage direction for pore 2 could be produced by a small interfacial barrier to charge entry into the pore, as previously described [17]. Thus, two contributions to rectification are proposed: one due to an interfacial barrier (as observed for pore 2) and the other the positively charged voltage sensor located on loops outside the transmembrane conducting pathway (the pore itself). The combined effects would be present in the rectification observed for pore 3. For pore 2, one would need to further propose that the positively charged sensor on the *cis* side of the membrane is the sole source of asymmetric charge that contributes to rectification. Perhaps, the sensor of pore 2 on the trans side is too far from the pore mouth to influence the rectification. If so, then the rectification of pore 2 only arises from the interfacial barriers to charge entry.

If the rectification directly observed at negative potentials with only pore 3 open is due to the charged voltage sensor located near the pore opening on the cis side, then trypsin cleavage of the sensor should reduce that rectification. Trypsin treatment on the cis side holding channel 2 closed to allow trypsin access to the sensor of channel 3 resulted in channel 2 reopening and loss of voltage gating, as expected, but also the loss of the rectification in the negative voltage region. Although this is not a clean experiment—because after trypsin treatment the current is flowing through both pore 2 and 3 and, in addition, trypsin might cleave at many sites—the loss of rectification is consistent with the proposed location of the voltage sensor and its dual role on both voltage gating and rectification.

Assuming that the positively charged sensor on the *cis* side of the membrane is the sole source of asymmetric charge that contributes to rectification, one can re-examine the rectification results. If all the pores have fundamentally equal properties, then all have the interfacial barrier effect. Therefore, to determine the rectification due to asymmetric charge, one must subtract, for each pore, the portion of the rectification due to the interfacial barrier. Based on the results for subunit 2, the interfacial barrier produces a 37 pS/100 mV rectification regardless of the sign of the applied electrical potential (from Table 4). In Table 5, that value is listed in column 4 after multiplication of the basic value by the number of open pores present.

By subtracting this from the measured total rectification (column 3), one obtains the rectification due to charge asymmetry (column 5). Note than when corrected for the number of charged sensors on the *cis* side of the membrane, all the values are consistent in that the rectification resulting from a positive potential on the *cis* compartment yields a rectification of −32 pS/100 mV (for one voltage sensor), whereas a negative potential yields a rectification of +112 pS/100 mV. Since the corrected values on column 5 are so remarkably consistent, the theoretical analysis seems to be justified.

### 3.4. Model of the Structure of Triplin That Embodies the Results Presented

**Figure 14:**
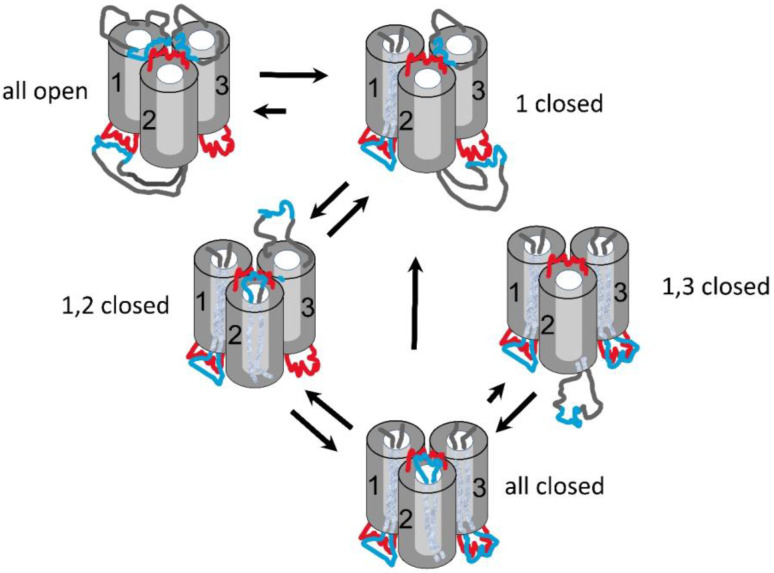
Revised model of the gating of Triplin based on the results presented. The top of the structure is the *cis* side of the membrane, the side from which Triplin inserted. The bottom of the structure is the *trans* side and that is the side maintained at virtual ground by the amplifier. All indicated voltages refer to the *cis* side. The numbers 1,2,3 refer to pores 1,2 and 3. Short arrows refer to reactions of lower probability. For simplicity, the closed state of the pore is illustrated as the result of blockage by a single loop of the beta barrel but, of course, multiple loops may be involved.

The results obtained were used to generate a model for the gating process (Figure 14). Based on the large conductance of the pores, it is likely that they are formed by beta barrels, similar to those formed by porins. Subunit 2 is oriented in the opposite direction to subunits 1 and 3. The sensor domain is proposed to be one of the loop regions of the barrel and its positively charged portion is illustrated in blue. A negatively charged complementary region (red) is proposed for each subunit, but no evidence exists for the existence of this complementary charge. Its presence stabilizes the closed state. In the fully open state, the sensor of subunit 2 interacts with one of the two negatively charged regions on either subunit 1 or 3 and is, thus, unavailable for cleavage by trypsin, as found experimentally. The positively charged sensor region of subunits 1 and 3 interact with the negative region of subunit 2.

The latter interaction is weak because of the repulsion of the two positive sensor domains from subunits 1 and 3. The application of a positive voltage on the *cis* side (top of the model) results in the closure of either subunit 1 or 3 and whichever closes is labeled as subunit 1. Closure is proposed to involve the entry of the loop region into the pore, thus, obstructing ion flow and resulting in a closed state. Such an intrusion of a loop region into a beta barrel is a feature of many porins [1]. Once the positively charged loop is in the pore, it interacts with the proposed negative region and is, thus, stabilized.

The closure of subunit 1 results in a tighter interaction between the sensor of subunit 3 and the negative region of subunit 2. Therefore, the sensor of subunit 3 becomes unavailable for reaction with anhydride or cleavage by trypsin. The closure of subunit 2 removes this interaction, allowing the sensor of subunit 3 to be available for reaction and gating.

### 3.5. Comparison of Triplin’s Mechanistic Insights with Insights into the Voltage Gating of Porins

Voltage gating in porins has been studied by many research groups [5,16]. Many have focused on loop 3, which is present within the conducting pore of some porins. A variety of changes in this loop can change the selectivity and conductance of the pores. This is expected because loop 3 is within the conducting pathway. Changes in loop 3 can affect the gating of the pores. Although sometimes referred to as changes in voltage dependence, these are changes of the voltage at which pores begin to close, not the typical meaning of voltage dependence that is the change in probability of being in the open state with change in voltage. More typically those changes in the voltage at which pores begin to close are referred to as changes in the critical voltage. Such changes could be the result of changes in the energetics or kinetics of the system, likely the latter, and are thus not very informative. For example, changes in the critical voltage were also observed when lysines in loop 4 and 6 of porin from Haemophilus influenzae type b were converted to glutamic acid [18]. These might be expected to have similar effects, as observed when using anhydride modification of Triplin. However, the results were not consistent with the modification of a voltage sensor. In some of these single point mutations, the critical voltage dropped to 50 mV. In some, the drop in critical voltage occurred mainly at positive potentials, while in others it was mainly at negative potentials, in another there were changes at both potentials and in still another there was no significant change. Thus, there was no logic in the outcome. Generally, a treatment that influences the critical voltage, whether it be a point mutation, a deletion, a chemical modification, a change in membrane lipid composition, a change in medium pH, or a change in ionic strength, does not give useful insight into the gating mechanism because the change likely resulted in a change in kinetics, and thus can have many causes. One can conclude that the altered region is somehow involved but not much beyond that. A further consideration that is often overlooked is that the high electric fields, often needed to achieve channel closure, cause the distortion of matter and this is quite different from discrete changes in conformation from one structure to another. There is also electrostriction of the bilayer membrane that is likely associated with changes in lateral pressure from the membrane onto any embedded protein. Thus, multiple effects place any interpretation on shaky ground.

With regard to pore closure with increasing voltage, the fundamental property on which to focus is the steepness of the voltage dependence, i.e., the change in open probability with changes in voltage. To achieve steep voltage dependence of the probability of a pore being in the open state, the energy level of the conformations of the protein must change with voltage. The only way that can happen is if a large amount of charge is moved through an electric field (Joules = Coulombs × Volts). For pores 2 and 3 of Triplin, 14 changes are the minimum required to account for the steep voltage dependence (more if the charges only move through a portion of the transmembrane voltage difference). Thus, the lateral motion of the l3 loop or exposure of hydrophobic residues to produce a vapor gap [16], etc., will not do. These may well be ways of closing a pore, but this closure would not be steeply voltage controlled.

### 3.6. The Physiological Role of Triplin

The physiological role of Triplin is currently just a matter of speculation. Its steep voltage dependence could allow *E. coli* to detect and respond to electric fields in the medium, such as those that exist especially near to local permeabilities in an epithelium, and thus the bacterium could enter the organism though the weakness in the epithelial wall. Alternatively, as pointed out by Nikaido and coworkers in 1988 [7], “membrane-derived oligosaccharides in the periplasm could quantitatively explain the magnitude of the Donnan potential”, which is needed to close even the weak-voltage gated channels, such as ompF and ompC, which only close at very high voltages. Thus, changes in the medium ionic strength or changes in the colloidal charges in the periplasm could gate Triplin with unknown consequences.

## 4. Materials and Methods

### 4.1. Materials and Handling of Reagents

All chemicals used were reagent grade. The phospholipids were obtained from Avanti Polar Lipids (Alabaster, AL, USA). Cholesterol, trypsin, and soybean trypsin inhibitor were purchased from Sigma (St. Louis, MO, USA). The succinic anhydride was dissolved in anhydrous dimethyl sulfoxide just before use at a concentration of 200 mM. Trypsin (7500 BAEE units/mg) was dissolved in 50 mM acetic acid at 1 mg/mL and stored frozen in 0.1 mL aliquots. Soybean trypsin inhibitor was made at a concentration of 10 mg/mL in water and stored frozen in 100 µL aliquots.

### 4.2. Electrophysiological Recordings

All experiments were performed on Triplin reconstituted in planar phospholipid membranes made from monolayers of phospholipids, as previously described [19,20]. Briefly, a teflon chamber was used consisting of 2 aqueous compartments separated by a thin polyvinylidene chloride partition containing a 0.1 mm hole. A planar membrane was generated across the hole from phospholipid monolayers formed on the surface of the aqueous compartments. This membrane is identical to a natural cell membrane but lacking in proteins or carbohydrates. The monolayers were formed from a solution of 0.5% (*w*/*v*) diphytanoylphosphatidylcholine, 0.5% (*w*/*v*) polar extract of soybean phospholipids, and 0.05% (*w*/*v*) cholesterol in hexane. The hexane is allowed to evaporate prior to membrane formation. The aqueous solutions consisted of 1.0 M KCl, 1 mM MgCl_2_, buffered with 5 mM PIPES pH 6.9 for rectification experiments, 50 mM HEPES, pH 7.8 for succinic anhydride experiments and 10 mM HEPES, pH 7.8 for trypsin experiments. The aqueous solutions on both sides of the membrane were identical in all experiments and each were 5 mL in volume. Samples containing Triplin were generated, as previously described [9]. These were stored at −80 °C in 0.1 mL aliquots after being flash frozen in dry ice/ethanol. After thawing, β-octyl-glucoside was added to one aliquot to a final concentration of 1% (*w*/*v*) and kept on ice during the experiment. Typically, 10 µL of the Triplin-containing solution was dispersed into aqueous phase on one side of the membrane and, with time, Triplin would insert into the membrane. The membrane voltage was clamped using a high-quality operational amplifier in the inverted mode and the current recorded using Clampex 10.3 software. Calomel electrodes were used to interface the aqueous phase with the electronics. The side of the membrane held at virtual ground by the amplifier was designated as the *trans* side, and thus the voltages reported are those on the opposite or *cis* side. The signal was low-pass filtered at 500 Hz. The sample containing Triplin was always added to the *cis* compartment.

### 4.3. Quantification of Voltage Dependence

This quantification was done for the succinic anhydride modification experiments. From the dependence of the equilibrium conductance of a channel former on the transmembrane voltage, one can extract 2 defining parameters: the number of charges (*n*) that must translocate through the entire voltage difference to account for the voltage dependence and the voltage at which the conductance is half maximal (*V*_0_). For the defining parameters to be limited to just 2, one needs to model the analysis to 2 conformational states: an open state and a closed state. Triplin forms 3 pores and each can be modeled, occupying the said 2 states; therefore, there are 8 possible states of Triplin. Since each pore-forming structure affects other pore-forming structures, the analysis needs to be limited to specific conditions. We limit it to two conditions. The gating of pore 1 cannot be analyzed because its gating kinetics are much too slow to measure the equilibrium conductance as a function of voltage within a reasonably achievable experimental time window. Gating of pore 2 can only be studied when pore 1 is closed and pore 3 is open (this is condition 1). Under this condition, the energetics of the structure forming pore 2 only depends on the transmembrane voltage. Although the current under this condition is flowing through both pores 2 and 3, only pore 2 gates at negative voltages and so the current through pore 3 does not influence the measurement of the voltage-gating parameters (see below). By subtracting the minimum conductance from the measured conductance (see Equation (3)), the conductance due to pore 3 is removed from the analysis. The second condition that was analyzed was the gating of pore 3. That gating can only be performed when both pores 1 and 2 are closed because pore 3 cannot gate otherwise. This takes place at positive potentials where pore 2 is typically closed, and thus not contributing to the measured current. Again, the energetics of the structure forming pore 3 only depends on voltage in this condition.

For the quantitation of both gating processes, the Boltzmann distribution was used (Equation (1)). The ratio of the probability of a pore being open to that being closed, *P_o_*/*P_c_*, is related to the free energy difference Δ*E* between those states.

(1) $$\frac{P_{O}}{P_{C}}=e^{-\frac{∆E}{RT}}.$$

where *R* is the gas constant and *T* is the absolute temperature. The free energy difference Δ*E* is divided into a voltage-independent term *nFV*_0_ and a voltage-dependent component *nFV*. *F* denotes the Faraday constant; *n* is the effective gating charge, i.e., the minimum number of elementary charges that move across the entire electric field upon closing; *V* is the applied voltage; and *V*_0_ is the voltage at which Δ*E* becomes zero, i.e., when half of the channels are open.

(2) $$∆E=nFV-nFV_{0}=nF\left(V-V_{0}\right)$$

The probability ratio, *P_o_*/*P_c_*, is obtained from channel conductance measurements following the common approach described elsewhere [21], so that:

(3) $$\;\ln(\frac{G_{max}-G}{G-G_{min}})=\frac{nF}{RT}\left(V-V_{0}\right),$$

The experimentally measured parameters, *G*, *G_max_*, and *G_min_* are the conductance at voltage *V*, the maximal conductance and the minimum conductance, respectively. Plotting the data according to Equation (3) allows one to obtain the parameter “*n*” from the slope of the line and *V_0_* from the intercept.

The Boltzmann distribution implicit in Equation (1) is valid at equilibrium, but in the experiments the voltage is changed in a linear fashion and so the kinetics of the system must be considered. Previous studies [9] demonstrated that the kinetics of pore closure for Triplin are very fast, whereas those of pore reopening are slow. Thus, only the conductance/voltage relationship from the closing process was used for the analysis. Slow, 30 mHz, triangular waves in which the voltage changed linearly with time (typically from −80 mV to +80 mV) were used to achieve an approximation to equilibrium conditions. However, as previously published [9], at this rate of voltage change, equilibrium is not quite achieved but approximated. Thus, the reported values of *n* underestimate the real values. Regardless, the same conditions were used before and after chemical modification, and so relative changes in the parameter n were obtained to look for changes in the number of charges on the voltage sensor.

### 4.4. Technique Used to Collect the Data and Overcome Stochastic Fluctuations

**Figure 15:**
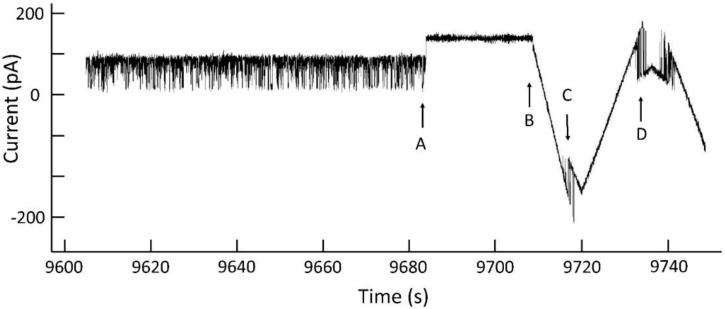
Subunit 3 oscillations between the open and closed states terminate when subunit 2 opens. The left part of the figure shows the oscillations of subunit 3 held at 34 mV. At point (A), subunit 2 opens, thus, locking subunit 3 in its open state. At point (B), a triangular voltage wave was applied. Subunit 2 closure took place in the region labeled (C) and subunit 3 closure in the region labeled (D).

When recording the current carried by a single pore, one observes the opening and closing of the pore from the rise and fall of the current. Thus, the state of that pore-forming structure is known unambiguously. However, single structures can use thermal energy to be open when their energy level would cause them to be closed and vice versa. Thus, the applied voltage and, therefore, the energy level of the structure only changes the ***probability*** of the pore to be open or closed. In Figure 2, the voltage at which pores 2 and 3 close varies with every cycle of the applied triangular voltage wave. By averaging many observations, one can obtain the fraction of time the pore is open at any given voltage. For experiments involving a single Triplin, we averaged the current records from at least 20 cycles of the triangular wave (i.e., 20 measurements or more) in order to reduce stochastic variability and obtain reasonable conductance–voltage relationships. Fewer records were averaged when multiple Triplins were present. For subunit 2, the informative part of the record was the current recorded when the voltage was changed linearly from high positive to high negative values (subunit 2 closure is a fast process). Subunit 1 was always closed in these records. For subunit 3, we used the current records from positive to negative voltage values (subunit 3 closure) but only those records in which subunit 2 remained closed or opened well after the closing of subunit 3. Based on the voltage range when subunit 2 closed, it should not be possible for subunit 3 to close because subunit 2 would always be open before the voltage reached positive values. However, subunit 2 often remained closed because of the kinetic delay in the opening of subunit 2. Note in Figure 2 the difference between the voltage at which subunit 2 closed and that at which it reopened. This hysteresis is the result of the kinetic delay in the reopening process. This requirement of subunit 2 closure in order for subunit 3 to close is also manifested in the inability of subunit 3 to close when subunit 2 reopens. In Figure 15, a single Triplin was present in the membrane, and the value of the transmembrane voltage was chosen so that subunit 3 would oscillate between its open and closed state. In this experiment, that value was +34 mV. After an extended period of time of subunit 3 oscillations at +34 mV, subunit 2 reopened (A) and subunit 3 could no longer close. The application of the triangular voltage wave (B) demonstrated that both subunits 2 and 3 were open because subunit 2 closed at negative potentials (C), followed by subunit 3 closure (D) and then reopening at positive potentials. Thus, the data collected for subunit 3 were always when both subunits 1 and 2 were closed. The data were collected in exactly the same way before and after succinic anhydride addition on the same reconstituted Triplin.

The use of voltage steps (rather than ramps) to collect data for conductance–voltage curves is problematic for large channels because keeping the channel at a fixed voltage for some time results in time-dependent adaptation to the voltage, which differs from one voltage level to the next, degrading the information that is obtained.

### 4.5. Statistical Analysis

Aggregate results are reported as an average ± the standard error of the mean. Significant differences in values were tested using the Student’s *t*-test.

## 5. Conclusions

The electrophysiological properties of Triplin only weakly resemble those of other pore-forming structures described in the literature, both those identified in prokaryotes and eukaryotes. The 3-pore structure, conductance, and ion selectivity most resemble ompF, but the combination of steep voltage dependence, intersubunit cooperativity, and likely antiparallel subunit organization is quite unique. The voltage sensor of each pore-forming structure was shown to be a highly positive structure whose translocation through the membrane is coupled to the gating of each of the pores. This mechanistically explains the source of the steep voltage dependence of the gating processes. Identifying the location and accessibility to the modification of the voltage sensor in different structural states also revealed how the closure of pore 1 is required for pore 2 closure and closure of pore 2 is required for pore 3 closure. This gives a mechanistic foundation for the unusual cooperative behavior. It is likely that the structure of the pores is a beta barrel, like that of the well-described porins. The voltage sensor is probably located in the loop regions at some distance from the pore opening as this is the best explanation for the weak non-linearity in the current/voltage relationship. The model of the gating process proposes that the loop(s) containing the voltage sensor enter the pore, block it, and, at the same time, translocate the charge effectively to the other side of the membrane. This gating mechanism differs from any other described for steeply voltage-gated channels but resembles the inactivation mechanism used by highly selective ion channels. However, in Triplin, the voltage sensor performs both necessary functions: it detects and responds to the electric fields and blocks the ion conducting pathway. This adds to the variety of gating mechanisms used by voltage-gated channels. In view of the many differences between Triplin and the porins described to date, Triplin may be a member of a new pore-forming family. The role of this complex machinery in bacterial physiology is unclear but considering its complexity and high sensitivity to small changes in transmembrane voltage, Triplin must be vital to some important function.

## Figures and Tables

**Table 1:** Properties of Triplin and those of some porins.

	Open Pore Conductance (nS) (in 1 M KCl)	Selectivity (Pc/Pa)	Pore Size (nm)	Number of Pores
Triplin	1.5	3.6	0.9	3
OmpC	1.5	26	1.0	3
OmpF	2.1	3.6	1.2	3
PhoE	1.8	0.3	1.1	3
LamB	2.7/0.2	4.5	1.4	3

**Table 2:** Accessibility of sensor to anhydride modification.

Subunit under Study	State of Subunit during S.A. Addition	State of Subunit 2 during S.A. Addition	Side of Membrane to Which S.A. Was Added	% Drop of *n*	# of Independent Experiments	*T*-Test *p* Value
2	open		*cis*	0.6 ± 4.1	7	0.43
2	open		*trans*	30 ± 5	12	0.00003
2	closed		*cis*	27 ± 6	12	0.0012
2	closed		*trans*	−5.1 ± 8.0	4	0.30
3	open	2 open	*cis*	5.7 ± 6.8	6	0.21
3	open	2 closed	*cis*	43 ± 6	10	0.00015
3	open	2 open	*trans*	2.1 ± 4.4	7	0.29
3	open	2 closed	*trans*	−7 ± 6	5	0.14
3	closed		*cis*	8.5	1	
3	closed		*trans*	49	2	

**Table 3:** Quantitative measurements of the rectification.

Subunits Open	Voltage Range	G/V (pS/100 mV)	Number of Experiments
1, 2, 3	positive	−176 ± 2	8
1, 2, 3	negative	+112 ± 4	9
2, 3	positive	−106 ± 4	11
2	positive	−36 ± 6	8
3	negative	+75 ± 4	11

**Table 4:** Rectification of individual subunits.

Subunit Open	Voltage Range	G/V (pS/100 mV)	Method Used
1	positive	−70	Subtracting (1 + 2 + 3) − (2 + 3)
1	negative	+75	Assuming rectification of 1 and 3 are the same for *V* < 0
2	positive	−36	From measurements
2	negative	−38	Assuming rectification of 1 and 3 are the same and subtracting (1 + 2 + 3) − 2(3)
3	positive	−70	Subtracting (2 + 3) − (3)
3	negative	+75	From measurements

**Table 5:** Rectification interpreted from the model.

Subunits Open	Voltage Range	Measured G/V (pS/100 mV)	Expected G/V If No Charge Asymmetry	Rectification Due to Charge Asymmetry (pS/100 mV)
1, 2, 3	positive	−176	−111	−65 (−32 per sensor)
1, 2, 3	negative	+112	−111	+223 (+112 per sensor)
2, 3	positive	−106	−74	−32 (−32 per sensor)
2	positive	−36	−37	+1
3	negative	+75	−37	+112 (+112 per sensor)

## Data Availability

All data available upon request.
